# A standardised equine-based welfare assessment tool used for six years in low and middle income countries

**DOI:** 10.1371/journal.pone.0192354

**Published:** 2018-02-21

**Authors:** Rebecca Sommerville, Ashleigh F. Brown, Melissa Upjohn

**Affiliations:** Brooke, Action for Working Horses and Donkeys, London, United Kingdom; University of Minnesota, UNITED STATES

## Abstract

The majority of horses, donkeys and mules (equids) are in low- and middle-income countries, where they remain a key source of labour in the construction, agriculture and tourism industries, as well as supporting households daily through transporting people and staple goods. Globally, approximately 600 million people depend on working equids for their livelihood. Safeguarding the welfare of these animals is essential for them to work, as well as for the intrinsic value of the animal’s quality of life. In order to manage animal welfare, it must be measured. Over the past decade, welfare assessment methodologies have emerged for different species, more recently for equids. We present the Standardised Equine-Based Welfare Assessment Tool (SEBWAT) for working equids. The tool is unique, in that it has been applied in practice by a non-governmental organisation (NGO) for six years across Low-Middle-Income Countries (LMICs). We describe the revision of the tool from an original to a second version, the tool methodology and user training process and how data collection and analysis have been conducted. We describe its application at scale, where it has been used more than 71,000 times in 11 countries. Case study examples are given from the tool being used for a needs assessment in Guatemala and monitoring welfare change in Jordan. We conclude by describing the main benefits and limitations for how the tool could be applied by others on working equids in LMICs and how it may develop in the future.

## Introduction

During the last few years there has been a rapid increase in tools available to assess animal welfare [1–4]. Researchers and practitioners have moved towards using animal-based measures for this purpose, as it was recognised that measuring human practices and resource management alone was insufficient, because animal-based health and behaviour measures best represent the status of the animal [5]. Groups of animals used for human purposes benefit from having their welfare assessed, in order to measure and manage their quality of life. Most tools for welfare assessment were originally created for animals in farms or laboratories, and specific tools for other species followed. This included animals used for work, which are still highly prevalent in many places. Nearly 100 of the 112 million equids (horses, donkeys and mules) worldwide are in Low-Middle Income Countries (LMICs) [6, 7]. The majority of these are working animals, who help approximately 600 million people worldwide [8], rather than being involved in sports or leisure activities. Owners and users of working equids (‘owners’) earn direct income generated by them via working in the agricultural, construction and tourism industries, as well as benefiting from support to households daily through transporting firewood, water, people or agricultural produce [9–11].

A variety of animal-based welfare assessment protocols have been developed and trialled in equids. The Animal Welfare Indicators project developed assessment protocols for horses [12, 13] and donkeys [14] as a follow up to the Welfare Quality® [1] project on farm animals. These could be used in equestrian sport disciplines and on farms [15, 16, 17]. Qualitative Behaviour Assessment has been applied to donkeys [18], although it has been suggested that this alone is not sufficient for a comprehensive welfare assessment because welfare is multi-faceted [19]. To document facial expressions across different contexts, Equine Facial Action Coding System was developed to record individual facial movements in different applications ranging from veterinary research to art and film animation [20]. The Horse Grimace Scale was also developed to identify equine pain responses, such as post-surgical pain [21] but does not apply to horses under other conditions and there is recognition that the face is not always the best place to look because pain involves other body areas too [22, 23].

Other aspects of equine welfare compromise have been explored through animal-based measures. Abnormal repetitive behaviour of equids has been described in detail, predominantly in horses, in relation to factors including the type of breed, feed, housing [24] and work [25]. Depression-like behaviour has been suggested to be visible in horses measured by a fixed gaze and head, ear and neck position, a flat posture and withdrawal from the surrounding environment [26]. Individual characteristics and extrinsic factors, such as housing conditions and environmental management, have been reported to influence equine welfare and learning abilities [27]. It has been shown that horse owners are not effective in identifying back pain [28] or abnormal repetitive behaviours via questionnaire when they are highly prevalent [29], highlighting the importance of using trained assessors and observation. A range of such measures have been brought together as tools to assess equine welfare, see Hockenhull and Whay [30] for a review.

All of these methodologies for welfare assessment were developed and applied predominantly in Upper-Middle Income Countries and High-Income Countries (HICs), because the majority of animal welfare funding and research is based there. In LMICs, where the majority of the world’s equids exist, the requirements for welfare assessment tools differ. A tool that focuses on issues typical to HICs (e.g. obesity, stereotypy) would not be suitable. Issues seen in the LMIC context rather than in HICs include lesions from hobbling (head collar restraint is not always the local norm), firing the skin (for identification or intended treatment) and mutilation to parts of the body (for cultural or identification reasons). Welfare issues that are generally more highly prevalent in LMICs than in HICs include gait abnormalities, low body condition, lesions from harnessing, fear of humans and poor hoof condition [23, 31]. Tools need to be practical to conduct with limited resources in difficult to reach locations. For example, welfare assessments may take place in remote communities that are only accessible by dirt road or track, at high altitude and there may not be electricity, running water, or internet access available. There may also be prevailing conditions of drought, extreme weather or political instability. A tool that takes a long time to perform or that requires more than very basic equipment would not be suitable.

Some welfare issues experienced by working equids in LMICs may affect a very high proportion of the population (e.g. 90–100% for lameness), substantially greater than in HICs [32]. As there is a risk that welfare problems can be perceived to be normal or acceptable, when they are highly prevalent in a population [28, 29, 33, 34], the welfare assessment approach needs to mitigate this. Methodologies for working equine welfare assessment need to detect the full range of relevant conditions. Whilst many of these are included in welfare assessment tools from HICs, issues may be less severe than in LMICs. Tools for equids in any setting need to capture the overall welfare status (from good to poor) and the severity and prevalence of individual issues. For example, a donkey welfare assessment protocol used in Botswana with 100 individuals [35] included an indicator for ‘Lesions and scars (number, location, severity)’.

The Standardised Equine Based Welfare Assessment Tool (SEBWAT) described here evolved from the Working Equine Welfare Assessment (WEWA) that was developed through a collaboration between Brooke, an international equine welfare Non-Governmental Organisation (NGO) and Bristol University [31]. To our knowledge, at the time it was the first animal-based welfare assessment tool to be used for working equids and to be applied in practice by an NGO. A condensed version of WEWA was applied to identify environmental risk factors across nine LMIC countries [36] and applied in Romania with different categorisation [37]. In LMICs, social research methods such as Participatory Rural Appraisal have also been applied to investigate equine welfare, by gathering from owners their perceptions of their animals’ welfare needs [38, 39]. Similar methods have been used to investigate risk factors for working equine welfare issues [32], which may be used instead of or alongside objective welfare assessment. Whilst participatory tools benefit from increasing engagement of owners with the outcome and resulting interventions at the community level, the accuracy of the welfare status captured could be lower. Trained assessors are required to record welfare information that is used to inform NGO, government, or industry policies and performance on working equine welfare.

Evidence generated from welfare assessment data is important for identifying welfare problems and evaluating potential solutions, tracking progress over time, supporting funding decisions, and conducting needs assessment, monitoring, evaluation and research. Governments, NGOs and other stakeholders interested in making progress on animal welfare require robust evidence in order to inform interventions. Welfare assessment data could be used to identify priority issues that need to be addressed through national policy initiatives or NGO programmes.

It is desirable that field-based tools in these contexts are: simple to learn to accommodate welfare assessors (‘assessors’) with potentially little formal education to be trained in the tool; very low cost, minimising equipment and technology because of the factors described which make assessment difficult such as limited technological infrastructure; rapid to conduct per animal to minimise the time owners are kept from work; using local resources of persons and equipment; acceptable to varying cultures for owners to agree to their animal being assessed; and transferable to different geographical and socio-economic settings to avoid needing adapting to each context.

Brooke is working to improve the welfare of working equids in LMICs in Africa, Asia and Latin America. Brooke operates by engaging communities to build their knowledge and change practices on equine welfare, providing emergency treatment and training to support the local animal healthcare provision infrastructure, and advocating for recognition and protection of equine animals through incorporation into national and international policies. This paper aims to present the SEBWAT for working equids and discuss the findings from its implementation by Brooke since 2010. We describe the development of the tool dating from a preceding version developed in 2003 (WEWA), the methodology, its application on a large scale in a real-life context on working equids, case examples of its use in Guatemala and Jordan, and conclude by discussing its strengths and weaknesses, the benefits for wider use, its limitations and potential future development.

### Development of the tool

Animal-based welfare data have been collected by Brooke from 2003 to date, the primary purpose of this collection is to identify prevalent welfare issues and to inform monitoring and evaluation of programmes. Monitoring typically involves annual data collection, the purpose of which is to track changes over time in a given region. The purpose of evaluation is to use historically collected data to inform the impact assessment of a project over a period of time, alongside assessment of the effectiveness of the structure and management of the intervention. Indirect benefits of using the tool include building the capacity of staff in equine behaviour and handling and generating a large database that can be explored to inform emerging needs, such as responding to disasters.

Since the initial conception and implementation of the WEWA tool, the tool needed to evolve to fulfil Brooke’s diversifying requirements for animal-based data, such as more sensitive measures to detect small changes over time, or differences between groups of animals for monitoring. Therefore WEWA was reviewed and replaced in 2010 with the tool presented here, SEBWAT, which has been implemented from December 2010 (the first assessment was in Jordan) up to the time of writing.

The review of WEWA was conducted considering its use in the preceding six years. Assessors from Brooke UK and international teams, external animal welfare scientists and veterinarians contributed to the review. This resulted in four key amendments: i) removing parameters with limited use (e.g. skin elasticity, side walk, cow hocks); ii) adding missing parameters (e.g. ear mutilations, ‘interference’ lesions from one hoof striking the adjacent limb or the hind hooves striking the heels of the fore limbs and genital lesions); iii) replacing or dividing binary scores into multi-level scores and iv) adjusting the sensitivity of measures of abnormality, which made the new tool more informative to Brooke.

For example, we compared WEWA and SEBWAT measures from data collected on 350 working equids in Petra, Jordan in November 2010. For eyes the abnormality indicator changed from binary (normal/abnormal) in WEWA to a three level (mild, moderate, severe) abnormality scale in SEBWAT [40]. With WEWA the result showed 100% prevalence of eye abnormality, whilst SEBWAT showed equids had no/mild abnormality (73%), moderate abnormality (13%) and severe abnormality (14%); therefore enhancing the sensitivity of the tool. Species comparisons revealed that with WEWA horses and donkeys/mules both scored 100% abnormality, whereas with SEBWAT horses had moderate abnormality (18%) and severe eye abnormality (9%), and donkeys/mules had moderate (11%) and severe abnormality (24%), therefore species-specific differences were revealed through the increased sensitivity. A further example of the effect of changing the rubric is for body lesions, whereby in WEWA lesions with fully broken skin but less than a minimum size of 4 sq cm would not be recorded, but would with the SEBWAT measure. Therefore Head/ears lesions (74%), Girth/belly lesions (55%), and Withers/spine lesions (81%) captured in SEBWAT were missed by WEWA. The adjusted measure sensitivity addressed underestimation of lesion prevalence in the population.

This demonstrates the benefit of welfare assessment protocols being periodically reviewed for the context they are used in to ensure that they are fit for purpose. Whilst here the tool changes applied to working equids, the review process could be applicable to other species’ animal outcome-based welfare assessments.

## Methodology of the tool

### Scoring criteria

SEBWAT comprises 40 animal-based measures and four descriptive identifiers, in addition to two optional fields of animal and owner identification, and a free text section for additional observations. Tables 1–6 describe the scoring for the parameters. S1 Appendix gives a full description of how each parameter is assessed.

**Table 1:** Scoring criteria for parameters of SEBWAT: Descriptors.

Parameter	Code	Description	Scoring criteria
Date	-	Date of assessment	N/A
Time	-	Time of assessment	N/A
Observer	-	Name of assessor	N/A
Region ID	-	Name or number of region	N/A
Animal ID	-	Name or number of animal	Determined by the local programme. Optional parameter
Owner ID	-	Name or number of owner	Determined by the local programme. Optional parameter
Work type	BKC	Transport of bricks by cart	Equid works in a brick kiln and pulls cart which contains bricks
	BKP	Transport of bricks by pack	Equid works in a brick kiln and carries bricks on its back in a pack, pack saddle, baskets or saddle bag
	TGC	Transport of goods by cart	Equid pulls a cart which contains items eg. food products (human or animal), stones, sand, water, wood, scrap metal, garbage, bricks (but the animal does not work in a brick kiln) etc.
	TGP	Transport of goods by pack	Equid carries items on its back in a pack, pack saddle, baskets or saddle bag eg. food products (human or animal), stones, sand, water, wood, scrap metal, garbage, bricks (but the animal does not work in a brick kiln) etc.
	ToC	Transport of tourists by carriage	Equid pulls a carriage or cart which carries tourists
	TPC	Transport of non-tourists by carriage	Equid pulls a carriage or cart that carries people who are not tourists
	ToR	Riding: Tourists	Equid is ridden by tourists
	R	Riding: Non-tourists	Equid is ridden by people who are not tourists eg for pleasure, sport, transport, riding school
	Ag	Agriculture	Equid is used in farming eg. ploughing, weeding, tillage
	Cer	Ceremonial	Equid is used during ceremonies such as weddings, funerals, religious festivals and any other social celebrations
	F	Foal	Equid has no adult teeth and is not used for work of any type. If the foal (or an animal with age Score 0) is working already, the code for the appropriate type of work should be recorded
	BT	Breeding or Trading	Equid (male or female) is used for breeding purposes, or bred for trading.
	O	Other	Equid is involved in any activity not included in the categories above eg. retired, orphan foal, ranch/herding horses, drawing water, kept solely for competition
Species	H	Horse	Species is *Equus caballus*
	D	Donkey	Species is *Equus africanus asinus*
	M	Mule	A hybrid of a donkey and a horse
Sex	S	Stallion	An entire male
	G	Gelding	A castrated male
	M	Mare	A female
Age group	0	Immature	Less than 3.5 years
	1	Young	3.5–7 years
	2	Mature	8–12 years
	3	Aged	More than 12 years

**Table 2:** Scoring criteria for parameters of SEBWAT: General health.

Parameter	Score	Description	Scoring criteria
Eyes	0	No abnormality	No abnormality in either eye, or only slight imperfections
	1	Moderate abnormality	Excessive tears or opaque liquid discharge extend beyond the corner of the eye or accumulated over the eyeball, red conjunctiva, or abnormal third eyelid is visible in one or both eyes
	2	Severe abnormality	Opacity, missing eye, eye fully or more than half way closed, clear swelling of eyelids or conjunctiva; abscess, ulcers, lesions, deformity on the eyeball or within 2cm around the eye; or blood-stained discharge is visible in one or both eyes
Mucous membranes	0	With normal range	The colour of the upper gum is within the normal range
	1	Paler than normal range	The colour of the upper gum is paler than the normal range or icteric
	2	Darker than normal range	The colour of the upper gum is darker than the normal range
Nasal discharge	0	None	No discharge, or only transparent liquid
	1	Discharge present	Opaque or blood-stained discharge is present in one or both nostrils
Respiratory noise	0	Not audible	Cannot hear breathing and there are no noises associated with breathing
	1	Audible	Can hear the animal breathing, or there are any respiratory noises when the animal inhales and/or exhales
Diarrhoea	0	None	No clear evidence of diarrhoea
	1	Diarrhoea present	Clear evidence of diarrhoea, or if diarrhoea is observed when defecating
Ectoparasites	0	None	No bot eggs, lice, lice eggs or ticks are seen on any part of the body
	1	Bot eggs	Any number of bot eggs are present on the animal
	2	Lice / lice eggs	Any number of lice or lice eggs are present on the animal
	3	Few ticks	One to five ticks are present on the whole animal
	4	Many ticks	More than five ticks are present on the whole animal
Body condition *(if all criteria for a full score are not attained*, *the half score below is awarded)*	1	Very thin	Neck concave; pelvis hollow; shoulder point, spine, ribs, hooks, pins and tail-head are prominent
	2	Thin	Neck concave or straight; pelvis flat; shoulder point, spine, ribs, hooks, pins and tail-head are visible
	3	Medium	Neck straight; point of shoulder not clearly visible and joins the body smoothly; spine slightly visible at withers but smooth elsewhere; ribs not visible; pelvis well filled and slightly rounded; tail-head slightly visible, but well filled and joins the rump smoothly
	4	Fat	Neck slightly convex; some fat accumulation behind shoulder; slight ‘gutter’ along spine; some fat accumulation over ribs; pelvis well rounded or slightly ‘heart-shaped’; some fat accumulation over tail-head
	5	Very fat	Neck distinctly convex; fat accumulation behind shoulder clearly visible; fat accumulation on either side of spine with a distinct ‘gutter’; fat accumulation clearly visible over ribs; pelvis distinctly rounded (clearly ‘heart-shaped’); fat accumulation clearly visible over tail-head

**Table 3:** Scoring criteria for parameters of SEBWAT: Behaviour.

Parameter	Score	Description	Scoring criteria
Observer approach	0	Positive	Equid is not afraid of the approaching observer, and is alert, friendly or relaxed, but not nervous or apathetic
	1	Negative non-reactive	Equid is apathetic, dull or non-responsive, and has no interest in the approaching observer
	2	Negative reactive	Equid is anxious, frightened or aggressive in response to the approaching observer
Chin contact	0	Accepts contact	Equid calmly allows the chin to be touched
	1	Avoids contact	Equid withdraws the head when contact with the chin is made, or as the hand is approaching the chin
Tail tuck *(donkeys only)*	0	No tail tuck	Equid does not show any signs of tail tuck whilst assessor is walking towards or around the hindquarters
	1	Tail tuck	Equid tucks the tail between the hind limbs, clamps down the tail, and/or tucks in or tenses the hindquarters at any time whilst you are walking towards or around the hindquarters
General attitude	0	Positive	Equid is not afraid, and is alert, friendly or relaxed, but not nervous or apathetic throughout the majority of the assessment
	1	Negative non-reactive	Equid is apathetic, dull or non-responsive throughout the majority of the assessment
	2	Negative reactive	Equid is anxious, frightened or aggressive throughout the majority of the assessment
Spinal contact	0	No reaction	Equid shows no clear reaction when contact with the spine is made
	1	Reaction	Equid shows visible tensing of the muscles of the back or neck, flinching of the part of the spine being touched, or clear movement of parts of the body other than the area being touched when contact with the spine is made

**Table 4:** Scoring criteria for parameters of SEBWAT: Body lesions.

Parameter	Score	Description	Scoring criteria*
Severity	0	None	No lesion in the specified Body Area, or there are only severity Score 1 lesions of less than the minimum qualifying size of 4 sq cm
	1	Superficial or healed lesion	Superficial or healed lesion, including hairless skin, which may be pale pink if partially broken, scabs, or scar tissue, greater than 4 sq cm
	2	Open lesion	Lesions where the skin and immediate subcutaneous layers are broken, including visible red tissue, dried or fresh blood, granulation tissue, lesions showing pus, or lesions which appear moist due to fluids seeping from the skin
	3	Deep lesion	Lesions deep enough to show muscle, tendon or bone
Size	0	None	No lesions, or the lesions affect less than 4 sq cm of the skin surface in the specified body area
	1	Small	Lesions affect between 4–16 sq cm of the skin surface
	2	Medium	Lesions affect between 17–64 sq cm of the skin surface
	3	Large	Lesions affect more than 64 sq cm of the skin surface

*For Body Area lesions severity and size are recorded. The ‘Body Areas’ are: head and ears, neck, breast and shoulders, forelimbs, withers and spine, ribs and flank, girth and belly, hindquarters, hind limbs. For lip, knee, genital or rectal, and tail or tail base lesions, only severity is recorded and there is no minimum qualifying size of 4 sq cm.

**Table 5:** Scoring criteria for parameters of SEBWAT: Practice-induced conditions.

Parameter	Code	Description	Scoring criteria
Mutilations: Tail, Ear, Muzzle	0	None	No mutilation of specified body part
	1	Healed	Mutilation of specified body part present, completely healed without broken skin
	2	Recent	Wound from mutilation of specified body part present, not healed with broken skin of any severity
Firing lesion: Body Areas*	0	None	No firing lesions on the whole animal
	1	One area	Firing lesions are present in one Body Area
	2	Few areas	Firing lesions are present in two or three Body Areas
	3	Many areas	Firing is present in more than three Body Areas
Firing lesion: Severity	0	None	No lesions on the whole animal
	1	Healed lesion	Healed lesions. This includes scar tissue (which may be hairless areas of white, pink, grey or black skin), and scars covered with white hairs
	2	Open lesion	Lesions where the skin and immediate subcutaneous layers are broken. This includes visible red tissue, dried or fresh blood, and granulation tissue
	3	Deep lesion	Lesions deep enough to show muscle, tendon or bone
Hobbling lesion: Severity		Horizontal hobbling lesions. Severity scored as for ‘Firing lesions’	

*The ‘Body Areas’ are as described for body lesions.

**Table 6:** Scoring criteria for parameters of SEBWAT: Hooves and limbs.

Parameter	Code	Description	Scoring criteria
Gait	0	Not compromised	Walks with even, regular strides, and ability to walk is not compromised. Motion does not need to be perfect
	1	Moderately compromised	Shows some irregularity or inconsistency of gait, and ability to walk is moderately compromised
	2	Highly compromised	Shows clear limping with every stride on the affected limb/s, but is able to bear weight
	3	Unable to bear weight	Severe lameness, and cannot bear weight on one or more limbs
Lower limb swelling *(Fore / hind)*	0	None	No swelling which clearly distorts the shape of the flexor tendons or fetlock joint on either of the fore limbs
	1	Swelling in one limb	Clear swelling in one limb
	2	Swelling in both limbs	Clear swelling in both limbs
Interference lesions *(Fore / hind)*		Lesions caused by brushing are found on the inner aspect of fetlock joints and pasterns, on fore limbs or hind limbs. Lesions caused by over-reaching are found on the heels of the fore limbs only. Severity scored as for ‘Firing lesions’	
Hoof shape *(One score for fore hooves*, *one for hind hooves)*	0	No abnormality	No or mild abnormality in both hooves
	1	One hoof abnormal	Clear abnormality in one hooves (toes too long or heels too long or low, wall concave or convex)
	2	Both hooves abnormal	Clear abnormality in both hooves
Hoof quality *(One score for fore*, *one for hind))*	0	No abnormality	No or slight hoof wall damage (nail holes, cracks or breakage affecting <2cm of hoof wall) in both hooves
	1	One hoof abnormal	Hoof wall damage in one hoof
	2	Both hooves abnormal	Hoof wall damage in both hooves
Frog condition *(Fore only)*	0	Frog not diseased	No evidence of disease of the frog in both hooves
	1	Frog diseased	Evidence of disease of the frog in one or both hooves (e.g. thrush, canker or discharge)
	2	Frog absent	Frog is mainly absent (e.g. eroded by disease, or severely over-trimmed)

### Sampling

Sampling strategies to use the tool on a subset of a population are designed according to the requirements of the region using it. Before 2015, in most contexts populations were sub-divided according to geographical location, animal work type and species and sampling was purposive in order to obtain welfare data from all relevant equine groups. Since 2015 in most locations and in some cases before, random sampling of individuals has been applied when possible. Logistical constraints of transport to remote areas, security, environmental conditions and limited resources, however, have meant that in practice convenience sampling is most used; with those individuals that are willing or most accessible being assessed. This introduces the risk of voluntary response bias, because owners that are motivated to attend a meeting may be more engaged, or conversely produce poorer welfare results if they anticipate potential free veterinary treatment to be provided there. These factors are considered during interpretation of data.

### Data collection protocol

The assessment takes five to ten minutes per animal. Data collection is conducted by two assessors working together, enabling assessors to agree on scoring, with the aim of increasing intra- and inter-observer reliability and repeatability. Typically, one assessor examines the animal and the other verifies and records the scores, and the pair is accompanied by an additional experienced animal handler. Assessors are encouraged to switch roles after assessing five animals to reduce the risk of observation error through fatigue. Assessors use a guidance manual which contains detailed descriptions and photographs on how to assign each score. A copy of this guidance manual is available on request from the corresponding author. The location of assessment is the animal’s usual place of work or accommodation, so is a familiar environment. Working equipment is either removed for assessment, or animals may be assessed in harness if it is safe and less disruptive to the animal to do so, but not if they are carrying a load.

### Training

Assessors complete a ten-day training course, during which they undertake theory and practical sessions on SEBWAT’s purpose and application, equine welfare, safe and positive reinforcement-based handling [41,42,43], and using the scoring criteria and assessment protocol. Competence in using SEBWAT is formally assessed via a written theory examination (pass mark 70%) and a practical standardisation test (pass mark 80%). Questions in the theory exam test theoretical knowledge, e.g. “describe the possible scores for lip lesions”; applying theory to photographs, e.g. “what score would you give this animal for age and why?”; and behaviour of assessors, e.g. “when assessing spinal contact, what should you do if the animal has a lesion on the spine where your fingers should be?”

Standardisation sessions are typically conducted with 15–20 equids, assessed individually by each trainee assessor under time restriction. Scores are subsequently compared with those of the trainer to identify discrepancies and trends for errors, and feedback about this given to trainees. Standardisation tests are intentionally conducted under stricter conditions than genuine field assessments, in which assessors can work in pairs without time restriction and have more experience of SEBWAT and equine handling associated with it. Assessors conduct periodic re-standardisation with a central trainer to maintain consistency and standardisation within and between geographical areas, typically biennially.

### Ethical statement

Ethical approval was not required as the assessment is non-invasive, observational and involves minimal disruption of animals and owners from their normal routine. Verbal informed consent is obtained from owners before commencing, when the purpose and nature of data collection is explained. Animals are restrained using a correctly-fitted head-collar or halter, or the animal’s own equipment/head-harnessing if safe and sufficiently comfortable to minimise disturbance. They are handled by an experienced, trained handler. The owner is present during assessment but does not directly interact with the animal after helping to prepare the animal, to reduce biasing human interaction parameters and to promote welfare and human safety. Data collection is non-invasive and only seven of the 40 animal-based measures require physically touching the animal. Required contact is restricted to opening the mouth, picking up fore-hooves and lifting the tail; all interaction is reinforced positively through touch, such as stroking the neck (known to reduce heart rate) [44,45], rubbing between the eyes and vocal praise. Food rewards are not typically used during assessment to avoid creating an expectation for the animal that treats will continue to be given after the assessment, or to owners that the NGO provides handouts. There are also practical barriers to transporting food to assess large numbers of animals. If the animal becomes distressed or shows signs of a highly aroused negative state then interaction causing the reaction is discontinued, but parameters which could be observed from a distance are still recorded and the animal reassured. This affects a minimal number of individual animals per data collection, but is not formally recorded. The sequence of measures (differing to their presentation in Tables 1–6) was specifically designed to be efficient and minimise disruption for the animal. On completion, assessors can provide feedback to owners on the welfare strengths and weaknesses identified and give advice on any action required, including referral for treatment if necessary. Animals requiring emergency treatment have this prioritised above welfare assessment; this is rare in practice. It is often necessary to gain permissions to work in the assessment areas, the nature of which varies according to the location and context. Examples include permissions from local chiefs or councils of village elders to work in a community, permissions from local government to work in particular districts, or permission from land owners to work on private land.

### Data entry and analysis

Three methods of data collection have been used. To begin, all data were collected manually on paper and then transferred into the in-house database on return to an office. This progressed in 2011 to some programmes using an electronic clipboard DigiMemo device using handwritten records, which saved time by removing manual data entry and potentially reduced human error. Finally, in 2014 a purpose-built Android Tablet application (Brooke Welfare Assessment Android Application) was developed and implemented in some countries. This allowed data to be uploaded electronically and removed the need for manual data entry. This increased accuracy compared to the electronic clipboard which depended on handwriting recognition and provided digital access to the guidance manual for assessors, rather than carrying bulky paper manuals.

Data are uploaded onto a bespoke central database, for the organisation to securely store and use them long-term; the database is accessible remotely through an intranet system. Descriptive data and prevalence percentages with confidence intervals are typically sufficient to use the results to inform the NGO programmes. On occasion, inferential statistics have been used to answer more in-depth research questions.

### Application of the tool at scale

SEBWAT records have been generated for 71,865 equids in 11 countries, across South Asia, East and West Africa, Central America, and the Middle East between December 2010 and December 2016. Data were utilised for a wide range of purposes, including needs assessment of the animals in new locations; prioritising which animal welfare issues to address; setting targets, monitoring and evaluation of intervention projects; identifying the type and prevalence of welfare issues in equine populations; mapping trends in welfare issues by work seasons, climate, work type, geographical area, owner demographic; and supporting field research, through providing data to inform research that considers the broader context around the animals. The tool has been regularly used on a grand scale. Tables 7–9 shows the number of assessments conducted in each country. This shows the geographical spread of LMIC locations where the tool has been applied and the large number of equids that have been assessed. It gives an example of the breadth of data collection and the opportunity to extract results in different ways, by showing the number of equids assessed using SEBWAT by type of work collected.

**Table 7:** Total number of SEBWAT assessments on working equids from 2010 to 2016 in countries listed by the work type of the animal: South Asia.

Country	Equine work category^1^	2010	2011	2012	2013	2014	2015	2016	Total
Afghanistan	Bricks	0	0	8	47	66	0	0	121
	Goods	0	50	163	94	200	0	97	604
	People	0	1	2	23	0	0	0	26
	Agriculture	0	102	359	524	763	0	637	2385
	Other	0	5	7	121	62	0	24	219
	**Total**	0	158	539	809	1091	0	758	3355
India	Bricks	0	1210	2894	3453	4412	2908	2202	17079
	Goods	0	505	1141	1737	1880	1480	1310	8053
	People	0	343	586	481	656	307	240	2613
	Tourists	0	20	226	0	287	4	82	619
	Agriculture	0	0	0	0	1	1	0	2
	Other	0	116	268	396	314	319	158	1569
	**Total**	0	2194	5115	6067	7550	5019	3992	29935
Nepal	Bricks	0	0	0	367	0	313	0	680
	Goods	0	0	0	335	306	376	0	1017
	People	0	0	0	442	1	488	0	931
	Agriculture	0	0	0	0	0	0	0	0
	Other	0	0	0	68	1	40	0	109
	**Total**	0	0	0	1212	308	1217	0	2737
Pakistan	Bricks	0	123	442	579	154	666	0	1964
	Goods	0	119	1255	1794	1086	1343	204	5801
	People	0	22	176	107	25	3	0	333
	Other	0	3	15	4	7	1	0	30
	**Total**	0	267	1888	2484	1272	2013	204	8128

^1^ Equine work categories grouped by Types of Work into: ‘Bricks’ (Transport of bricks by cart, Transport of bricks by pack), ‘Goods’ (Transport of goods by cart, Transport of goods by pack), ‘People’ (Transport of non-tourists by carriage, Riding), ‘Tourist’ (Transport of tourists by carriage, Riding: Tourists), ‘Agriculture’ (Agriculture), and ‘Other’ (Ceremonial, Foal, Breeding or Trading, Other, Not Observed).

**Table 8:** Total number of SEBWAT assessments on working equids from 2010 to 2016 in countries listed by the work type of the animal: Africa and Middle East.

Country	Equine work category^1^	2010	2011	2012	2013	2014	2015	2016	Total
Jordan	Goods	7	0	0	0	0	0	0	0
	People	20	0	0	0	0	0	0	0
	Tourists	308	0	0	0	0	0	0	0
	Agriculture	0	0	0	230	0	0	238	468
	Other	16	0	0	2	0	0	13	15
	**Total**	351	0	0	232	0	0	251	834
Ethiopia	Goods	0	0	0	217	149	0	1632	1998
	People	0	0	0	96	38	0	407	541
	Other	0	0	0	0	0	0	114	114
	**Total**	0	0	0	313	187	0	2153	2653
Kenya	Bricks	0	0	1	0	0	0	0	1
	Goods	0	548	1500	192	2951	4313	4245	13749
	People	0	0	0	0	0	13	3	16
	Tourists	0	0	1	0	0	0	0	1
	Agriculture	0	0	0	0	3	5	4	12
	Other	0	27	74	2	87	105	144	439
	**Total**	0	575	1576	194	3041	4436	4396	14218
Senegal	Goods	0	0	0	0	388	64	145	597
	People	0	0	0	0	61	81	112	254
	Agriculture	0	0	0	0	45	237	223	505
	Other	0	0	0	0	11	17	17	45
	**Total**	0	0	0	0	505	399	497	1401

^1^ Equine work categories grouped by Types of Work into: ‘Bricks’ (Transport of bricks by cart, Transport of bricks by pack), ‘Goods’ (Transport of goods by cart, Transport of goods by pack), ‘People’ (Transport of non-tourists by carriage, Riding), ‘Tourist’ (Transport of tourists by carriage, Riding: Tourists), ‘Agriculture’ (Agriculture), and ‘Other’ (Ceremonial, Foal, Breeding or Trading, Other, Not Observed).

**Table 9:** Total number of SEBWAT assessments on working equids from 2010 to 2016 in countries listed by the work type of the animal: Central America.

Country	Equine work category^1^	2010	2011	2012	2013	2014	2015	2016	Total
Guatemala	Bricks	0	0	0	0	0	1	0	1
	Goods	0	470	1594	890	1622	1066	96	5738
	People	0	83	272	169	202	31	0	757
	Tourists	0	2	15	0	0	0	0	17
	Agriculture	0	0	0	0	0	0	0	0
	Other	0	33	119	63	133	90	3	441
	**Total**	0	588	2000	1122	1957	1188	99	6954
Mexico	Goods	0	0	0	0	0	0	87	87
	**Total**	0	0	0	0	0	0	87	87
Nicaragua	Bricks	0	0	0	0	0	0	0	0
	Goods	0	0	300	0	104	330	372	1106
	People	0	0	126	0	10	113	122	371
	Tourists	0	0	27	0	0	0	0	27
	Agriculture	0	0	3	0	3	0	0	6
	Other	0	0	7	0	3	3	38	51
	**Total**	0	0	463	0	120	446	532	1561

^1^ Equine work categories grouped by Types of Work into: ‘Bricks’ (Transport of bricks by cart, Transport of bricks by pack), ‘Goods’ (Transport of goods by cart, Transport of goods by pack), ‘People’ (Transport of non-tourists by carriage, Riding), ‘Tourist’ (Transport of tourists by carriage, Riding: Tourists), ‘Agriculture’ (Agriculture), and ‘Other’ (Ceremonial, Foal, Breeding or Trading, Other, Not Observed).

### Case example one: Situation analysis and needs assessment in Guatemala

SEBWAT data were collected by Equinos Sanos para el Pueblo (ESAP, known as Healthy Equines for the People) in the eastern dry corridor of Guatemala in June 2015 as part of a situational needs assessment of the welfare state of working equids in the departments of Zacapa and Chiquimula, which were new to the organisation. This region is arid, prone to drought and its inhabitants are largely dependent on subsistence farming and agricultural labour for their livelihood. Equine population information was extracted from a 2003 census by the Ministry of Statistics, although this was recognized to be out of date. All animals that attended a pre-announced meeting in each community with ESAP were assessed. Further animals found during transect walks around each community were also assessed. The experience highlighted one of the challenges of sampling in this kind of remote context, where many homes are widely dispersed in areas with limited access due to the hilly terrain and dirt roads, and the availability of owners and their working equids varied, requiring convenience sampling of meeting participants. Further welfare issues were captured by talking to owners; these included castration and soft palate removal procedures performed without anaesthesia or analgesia. Four hundred and two animals were assessed in 18 communities across the following municipalities of the department of Zacapa: Huité (26% equids assessed / equid population estimate provided by community 98 /380), Estanzuela (21%, 21/100), Cabañas (7%, 9/130), San Jorge (72%, 163/225), Zacapa (34%, 85/250), and two communities in the department of Chiquimula (19%, 26/135). The most severe or prevalent issues across all animals sampled were body condition (41% underweight), hoof shape (41% abnormal in all limbs), gait (38% highly compromised), and adverse response to spinal contact (33% responded).

The results contributed to informing an evidence-based decision for Brooke to fund work by ESAP in the dry corridor region from 2016. By enabling the level of need of the animals in comparison to other locations including programmes funded in Nepal, Jordan and Guatemala; a decision was supported on how to allocate Brooke’s resources globally. ESAP withdrew from other locations in Guatemala where interventions were complete and moved into these two new departments. The most prevalent welfare issues were identified and issues set out by community so that local interventions could be tailored. Other data used to inform the decision included the most recent equine population census and population data from the communities, and information gathered by ESAP on the clinical status of the equine animals and an owner questionnaire in separate visits to the same communities after conducting SEBWAT.

### Case example two: Monitoring welfare change over time in Jordan

SEBWAT data were collected on three occasions over a period of six years by Brooke in Petra Archaeological Park and surrounding areas of Wadi Musa town and Um Sayhoun village, at the animals’ usual places of work or accommodation. All animals were engaged in work within the tourism sector, transporting tourists within or around Petra Archaeological Park. Table 10 describes results from SEBWAT on the hoof shape and hoof quality parameters, over three points in time for each group. SEBWAT data were collected by the same observer, always during the low tourist winter season. In 2010 there were 290 animals assessed, a purposive sample of 128 / population estimate by Brooke staff of 320 riding horses (40%), 26 / 30 carriage horses (87%) and 136 / 250 riding donkeys or mules (54%). In 2013 there were 232 animals assessed, 96 / 300 riding horses (32%), 27 / 27 carriage horses (100%) and 108 / 250 riding donkeys or mules (43%). In 2016 there were 204 animals assessed, 67 / 100 riding horses (67%), 27/ 27 carriage horses (100%) and 110 / 200 riding donkeys or mules (55%).

**Table 10:** Percentage prevalence of equids in Petra, Jordan with abnormal hoof shape or quality in fore and hind limbs in 2010, 2013 and 2016.

Equine group	Limbs	Hoof shape (%)			Hoof quality (%)		
		2010	2013	2016	2010	2013	2016
Riding horses	Fore	37	28	4	37	13	12
	Hind	14	8	6	31	12	4
Carriage horses	Fore	38	30	4	50	37	11
	Hind	8	4	4	46	4	7
Riding donkeys and mules	Fore	18	5	1	26	3	4
	Hind	12	2	3	13	0	2

Results indicated sustained improvement in hoof shape in fore and hind limbs over time, with negligible abnormality recorded in all three groups by 2016. The data also indicated improved hoof quality between 2010 and 2016, with all three groups recording a low prevalence of abnormality by 2016. Donkeys and mules were all unshod, and the majority of riding and carriage horses were shod with the standard u-shaped metal shoes, which helps to explain the species differences in hoof parameters in Table 3. Training owners was amongst interventions conducted by Brooke, and riding and carriage horses were serviced by Brooke-trained farriers. In April 2010 Brooke handed over its free veterinary clinic located at the Petra park to the Jordan Ministry of Agriculture to encourage the sustainability of the service. The quality of the government and private health services were targeted instead, through building capacity of government vets, improving clinical supplies with the ministry and supporting improved quality of the Horse Owner Association farriers. A tourism campaign ‘Care for Petra’ was also run with tourist industry representatives to influence tourist’s behaviour to support good treatment of the equids being used in the Petra park. The reduction in hoof abnormalities over time indicates improved farriery practices and owner use of the available services may have contributed to the reduction in hoof abnormalities over time.

The reduction in hoof abnormalities over time indicates improved farriery practices and owner use of the available services may have contributed to the reduction in hoof abnormalities over time, however this cannot be attributed without a case controlled study to exclude other factors. These SEBWAT parameters have contributed evidence to support programme decision-making, for example for Brooke to withdraw direct intervention in Jordan due to sustained welfare improvements when Brooke’s intervention had been scaled back.

## Discussion

The WEWA [31] was one of the first equine welfare assessment tools to be developed in a LMIC context and following its update to SEBWAT has been applied for six years internationally across 11 countries, continuing to be used in more. To our knowledge, this has been the most widely applied standardised welfare assessment for equine animals in LMICs.

### Validating measures of equine welfare

Before equine welfare can be measured, it is necessary for individual indicators to be validated. A body of research continues to identify and evaluate suitable measures of welfare [30]. These span the domains of welfare of nutrition, health, environment, behaviour and mental states [46]. Within the health domain, measures for welfare assessment have included lameness [47], signs of illness [23] and back disorders [28]. Whilst Broster’s study [47] was conducted in LMICs, Hausberger et al’s [23, 25] and Lesimple et al’s studies [28, 29] were completed in HICs. SEBWAT is based on recording observable measures rather than a clinical diagnosis. Therefore disease diagnoses, such as clinical lameness, infectious diseases, or heat stress are not captured in the tool, unlike Luna et al’s [48] that comprises a clinical examination. Within the environment domain, measures have included heat stress and drinking water temperature [49, 50], in a study in a LMIC. Pritchard et al’s study [50] investigated validation of skin elasticity indicators of dehydration against clinical pathology and concluded that skin elasticity was an unreliable indicator for use in the field. This indicator was therefore removed as part of the transition from WEWA to SEBWAT. Within the behaviour domain, measures have included abnormal repetitive behaviour [24, 25, 29], play [51] and Qualitative Behavioural Assessment [18]. Within the mental states domain, measures have included gregariousness, nervousness, fear [27], pain [52], depression and inactivity [26]. Beyond the animals themselves, the socio-economic, environmental and human behaviour factors that influence equine welfare have been studied, such as attributes of owners [53]. SEBWAT and other tools have been informed by these validation studies, which provide practitioners with the knowledge of reliable welfare indicators.

### Equine welfare assessment in high income countries

Several welfare assessment protocols have been developed and applied in HICs which combine different measures into a tool, with indicators from different domains forming an overall welfare assessment of an individual or group. In HICs there are typically more resources available, such as behaviour monitoring technology used in other species [54]. The AWIN protocol for horses [12] and donkeys [14] is one example of an HIC assessment applied to equine animals in sport, leisure [16] and on farms [15]. It is shorter to complete than its predecessor Welfare Quality® [1], which was also not validated for equids. The Horse Welfare Assessment Protocol (HWAP) [17] is a further HIC example, piloted on leisure riding horses. It benefits from combining animal, resource and environment-based measures and identifying risk factors for poor welfare. The measures had high repeatability, however perhaps due to the scale of the tool only 22 animals can be assessed by one person in a working day. With SEBWAT a pair of assessors can assess up to 60 animals per working day, though as with any tool the cost, transport time to sites and disruption to owners’ work schedule require consideration. SEBWAT training involves a substantial one-off expense, to cover the cost of the trainer, trainees’ time away from their normal role, as well as travel and accommodation costs when applicable. Where staff turnover and programme size permits, this expense can be offset by building capacity of in-country teams to train their own assessors, rather than requiring an international trainer. This has been achieved in several Brooke country programmes, but depends on retention of the experienced trainers.

### Equine welfare assessment in low middle income countries

In contrast to HICs, welfare assessment tools must be tailored to take account of low resource availability and local context constraints to be used in LMICs. They are used for a wide range of purposes, from community engagement in animal welfare, to monitoring of programmes, to welfare needs assessments, and tools may be adapted to meet these different aims. They must be quick to conduct to minimise the time owners and their animals are away from their work, to increase the chance of them agreeing to participate. They should also have a very low variable cost other than assessor time. It should also be noted that the quality of available and accessible equine appropriate healthcare may be lower than in HICs [55], which means that there can be a lack of diagnostic tools to investigate issues once identified, or professionals trained to treat an issue. SEBWAT was developed with these needs at its core. The only equipment needed is a head collar and rope, clipboard or tablet device to record data and a hoof pick. It can be taught and standardised with anyone who is literate and willing to safely and compassionately handle animals; assessors do not require a scientific or veterinary background. There are no physiological measures, so that it is not limited by local availability of equipment or laboratory services to test clinical samples. It is quick to conduct in less than 10 minutes per animal.

As with HIC assessments, SEBWAT results must be translated for lay audiences, including owners, due to its technical complexity. Assessment by an outside person risks reduced engagement with the findings more than if owners are able to participate and do an assessment themselves, e.g. [56]. Brooke’s Participatory Animal Welfare Needs Assessment [57], for example, is a tool used to engage communities with their animals’ welfare rather than to provide standardised data. With SEBWAT, real-time feedback is provided to each owner about their animal; some programmes combine positive feedback with a suggested aspect to improve for each owner. The time delay for full data analysis and returning on later visits, however, potentially hinders engagement with the wider community as compared with more participatory approaches.

Welfare assessments in LMICs should enable organisations to be responsive to the local country, socio-economic contexts and community needs, and to allocate resources effectively. A condensed or re-categorised version of WEWA was applied across Afghanistan, Egypt, Ethiopia, Guatemala, India, Jordan, Kenya, Pakistan, The Gambia [36], Romania [37, 58] and Egypt [59]. Since 2010 SEBWAT has been used in many of these locations again and Senegal, Mexico, Nepal and Nicaragua in addition. The breadth of its indicators means it can be applied to different contexts without adaptation; conversely they could be reduced because not all indicators apply in all settings. In some cases, particularly the midpoint of projects, a subset of the SEBWAT indicators has been used to collect data only on the measures which are the focus of an intervention. Data from some measures have proved more useful than others; some have been globally useful (e.g. descriptors: age, species, sex, work type; body condition; lesion severity; observer approach, general attitude), whilst others have been specific to certain contexts (e.g. ectoparasites, firing, mutilations) and a few have rarely been prioritised or found central to programme decision making (e.g. size of lesions, tail tuck, respiratory noise). In examples of tools by others, direct observation, clinical examination and an owner interview were combined in Chile to assess urban draught horses [48]. The involvement of direct indicators and indirect indicators strengthens assessment; however clinical examination requires a veterinarian, which limits a tool’s wider applicability. The ‘Hands-On Donkey Tool’ is a LMIC example that was applied in Mexico [60]. It is less comprehensive which means there are less measures to collect and it has practical application to improve welfare. Some measures (e.g. the composite behaviour and ill health score), may require further validation in comparison to other existing tools.

A further consideration for welfare assessment in LMIC contexts is that training for assessors typically requires knowledge and skills in welfare in order to use a tool, since these may not have been taught during previous education. For SEBWAT training usually lasts 10 days, or 5 days using blended learning with the theory preparation in advance. The practical component of training includes animal handling, in contrast to most farm welfare assessment training. Handling animals during assessment enables some parameters to be observed more accurately and enables assessors to set positive handling examples with owners. The limitation is that training takes longer. Following training, tools must be used regularly to maintain these skills, which is typically at least once a year for SEBWAT. Assessors must reach 80% agreement with the SEBWAT trainer to pass and periodic re-standardisation takes place to use the tool.

### Use of SEBWAT to inform field work and assess impact

The main purpose of welfare assessment tools is to assess and ultimately improve animal welfare, though they are used for other purposes such as legal compliance. Welfare assessment data should contribute to monitoring, evaluation and impact assessment of animal welfare programmes, whether they are NGO, government, or industry-led. This function is well established in human development organisations, but so far has been limited in the animal welfare sector [61]. How welfare assessments are applied and used to inform change is a key stage in the welfare improvement process, but is currently under represented in published literature. SEBWAT has and continues to provide animal-based data on major welfare issues of working equids, to determine the scale of the issues and prioritise whether interventions are required. In combination with other tools, SEBWAT findings have informed decisions around what type of interventions were required based on the issues. Farriery interventions were highlighted as a requirement in Ethiopia and Jordan due to a high prevalence of hoof problems, for instance.

Data collected by NGOs should be used to inform research questions due to its practical relevance and insights, as has been done for SEBWAT by field research programmes [62,63] and universities. Welfare assessment data can also be used to inform briefings for policy makers at governments and institutions. For example, SEBWAT data informed the discussion of environmental, human labour and animal welfare concerns in the South Asian brick kiln industry [64]. The implementation of the World Organisation for Animal Health Terrestrial Animal Health Code chapter on Welfare of Working Equids [65] could also use welfare assessments to identify priority issues. As well as pre-planned programmatic work, welfare assessment data can also usefully inform interventions by NGOs to address both slow-onset problems such as drought, as well as emergencies such as earthquakes or flooding. They can be used to support monitoring of interventions to address the secondary effects of political instability which for example, may result in reductions in income generated by animals used in tourism and their owners’ capacity to afford to buy animal feed. Examples of the use of SEBWAT for these situations have been generated in Afghanistan, Egypt, Senegal and Nicaragua. Evidence from historical datasets can be compared to the current conditions of the equids and new assessments carried out to gather the extent of need, for example to inform decisions to provide emergency feed and water according to body condition.

### SEBWAT limitations

Limitations of SEBWAT include that technical knowledge and skills are required for data handling and interpretation. Time is required for data handling subsequent to data collection, during which there is risk of human error if this is done manually rather than using a tablet application. Data management does not have to be performed by assessors, but further training specific for this may be useful to improve engagement of assessors with the findings and to assist with context-appropriate data interpretation. Large scale data collection is demanding within the often challenging environmental conditions and due to the requirement for animal handling, therefore assessors need to be physically fit. SEBWAT results are recorded at one brief moment in time and do not capture the duration of an experience or events at other life stages, therefore they do not inform Quality of Life or cumulative lifetime experience [66, 67]. Assessing quality of life requires steps beyond welfare assessment, where information from different sources should inform a practitioner’s opinion, especially for treatment decisions [68]. For example, SEBWAT does not capture what happens at the end of an equid’s life, yet the methods of slaughter [69], euthanasia [70] and abandonment [71] are serious welfare concerns for working equids.

As an animal-outcome based tool, omissions of SEBWAT include issues related to resource inputs and human behaviour, as well as details of the root causes of the welfare issues. Therefore SEBWAT results should be triangulated with data from other sources. These are identified through separate community engagement sessions and tools on Owner Behaviour Monitoring [72], Animal Healthcare Mentoring [73] and anecdotal observations by assessors. A combined overall welfare score is not made within SEBWAT due to the challenge of objectively weighting different measures against each other and the loss of detail that informs field-based decisions, although other welfare assessments tools have done this [67]. Such weighting involves an ethical value-based, rather than scientific judgement [74].

### Use of welfare assessment tools to inform positive change

The importance of positive welfare is increasingly recognised, as the sector recognises that animals require positive experiences for a good life, beyond minimising suffering [75]. Welfare assessments should aim to incorporate this change of approach wherever possible. Due to scientific opinion at the time it was created and its purpose to measure equine welfare in LMICs, the focus of SEBWAT is predominantly on negative aspects of equine welfare. Although some positive aspects are captured, such as alert and relaxed behaviour in response to an observer approach or general attitude and healthy body condition score, most measures are negative, or the absence of a negative result. Positive opportunities or choice can be measured through preference testing of animals [76]. Due to the difficulty of measuring animal affective states, limited measures available [77] and the prevalence of health-related issues, the majority of indicators in current welfare assessment tools, including SEBWAT, are negative, physical indicators of welfare. All welfare assessment tools should be regularly reviewed and updated for their validity, practicality and usefulness to inform positive change in the local context, whether for LMICs or HICs.

## Conclusion

SEBWAT has been developed and applied on the largest scale known to date of any equine welfare assessment tool, and is unique in that it has been widely applied in LMICs. The tool may be useful to others working in LMIC contexts with working equids, who wish to identify key welfare issues, track changes over time or reveal associations between different aspects of welfare with the surrounding environment. Results have been useful to inform an NGO’s interventions, monitoring, evaluation and impact assessments, as well as scientific research and government and institutional policy. In practice, the tool is quick to use per animal and comprehensive in content, but it reflects some of the barriers applicable to other welfare assessment tools through requiring intensive training to carry out, and analysis requires an understanding of data. SEBWAT focuses solely on the animal, which benefits from capturing animal outcome-based data, but therefore requires additional effort to triangulate the results with owner- and resource-based data and to engage the owners in their animal’s welfare. SEBWAT focuses on select negative, physical and behavioural indicators of equine welfare and does not capture all the factors that affect welfare throughout an equid’s life. Therefore it may need to evolve again in the future to reflect emerging science on positive animal welfare and cumulative life experience, but will crucially need to retain its practicality and usability for different contexts, including LMICs, to remain useful.

## Supporting information

S1 Appendix Assessment protocol for each parameter. (DOCX)

S1 Dataset (XLSX)
