# Supplementary material for: A standardised equine-based welfare assessment tool used for six years in low and middle income countries
Source: PLoS One. 2018 Feb 21;13(2):e0192354. doi: 10.1371/journal.pone.0192354 (PMC5821349; doi:10.1371/journal.pone.0192354)
Supplement: S1 Appendix — (DOCX) [file pone.0192354.s001.docx]

**S1 Appendix.**

**Assessment Protocol for each parameter (see Table 1 in paper for scoring criteria)**

**Table 1a. Descriptors**

| **Parameter** | **Assessment protocol** |
| --- | --- |
| Date and Time | - Record the date of assessment in the format day/month/year. Record the time of assessment in 24 hour clock format. |
| Observer | - Record the initials of the person carrying out the observations, not the person recording the data. |
| Region ID | - Record the code which identifies the region where the assessment is occurring. |
| Animal ID | - If you have a code for each animal, record it here. This is optional. |
| Owner ID | - If you have a code for each owner, record it here. This is optional. |
| Work type | - Record the code for the type of work done by the animal. If the animal does not fit into any of the work type categories, record work type as ‘Other’ and make a note of its work in ‘Other observations’. - If the animal does more than one type of work, use the following to help you decide which category it belongs in. If the animal does different types of work in different seasons, record the work type which the animal is doing in the current season (when the assessment is taking place). If the animal does different types of work throughout the day/week/month, record the work type which the animal does for the majority of the time. If the animal does more than one work type equally, record the work type which the owner bought it to do. If you are still undecided, record the work type the animal is doing at the time of assessment. If the animal does more than one work type equally and is not working at the time of assessment, then decide on one work type to record and make a note that the animal is equally engaged in other work types in ‘Other observations’. If there are many animals who do the same combination of work types you should try to record them all in the same way e.g. record all those animals as the same work type and make a note of the other work types in ‘Other observations’. |
| Species | - Record the species of the animal using the appropriate code. |
| Sex | - Record the sex of the animal using the appropriate code. A stallion is a male animal which has not been castrated., a gelding is a male animal which has been castrated (testicles removed), a mare is a female animal. |
| Age group | - Ageing of equids is done by examining the incisors (front teeth), and considering the following features: i. whether the incisors are temporary or permanent, ii. the angle of occlusion, iii. the shape of the tables of the lower incisors, iv. the markings on the tables of the lower incisors. - Use a combination of information from all of these features to make an approximate estimation of the age. As you examine the mucous membranes, gently open the lips to look at the animal’s incisors from the front and from the side. - Remember to look at both sides of the mouth. You may need to open the mouth to see the tables of the lower incisors. Your fingers should be placed in the diastema, and not between the incisors, canines or premolars. If the animal shows resistance to opening the mouth, do not continue. - If you have not seen enough of the teeth to decide on the appropriate score, record ‘Not observed’. |

**Table 1b**. General Health

| **Parameter** | **Assessment protocol** |
| --- | --- |
| Eyes | - Look at both eyes for abnormalities, do not touch them or the surrounding area. If there is a combination of symptoms from the scoring categories, record the highest score present. |
| Mucous membranes | - Observe the colour of the mucous membranes of the upper gum. If the animal shows resistance to opening the mouth, do not continue, and instead record ‘Not observed’ for this parameter. - There is a gradual spectrum of colours between normal and abnormal, therefore it can be difficult to decide which score to give to borderline colours. Make the best estimate you can based on the information available. |
| Nasal discharge | - Look at both nostrils for discharge, do not touch them. Record any amount of opaque liquid discharge. This may be white, yellow, green or blood-stained. |
| Respiratory noise | - Listen for audible breathing and respiratory noise whilst assessing the other parameters throughout the welfare assessment. - If the background noise in the environment is so loud that you feel you cannot hear whether the animal is breathing audibly or not, record ‘Not observed’. |
| Diarrhoea | - Observe inside the thighs and the back of the hocks for signs that the animal has diarrhoea. Ignore the area immediately around the vulva (in females) and perineum. - Record the presence of any amount of fresh or dried diarrhoea. - Ignore general staining, mud, pieces of fibre or solid material, or discolouration of the coat. There should be clear evidence of diarrhoea. |
| Ectoparasites | - Ectoparasites are organisms which live on, or feed from the equid from outside of its body. Look for bot eggs, lice, lice eggs or ticks on the whole body of the animal, on both sides. - Gentle contact with the finger is allowed to help you decide whether an object is an ectoparasite or other debris, e.g. mud. Do not pull the hair or the ectoparasites. - Do not record clinical signs, e.g. hair loss, itchiness, scratching. Be sure to look in particular at the ears, along the mane, under and around the tail, and behind the elbows. |
| Body condition  *(If all criteria for a full score are not attained, the half score below is awarded)* | - Body condition score is an estimation of the fat and muscle coverage on the animal’s body. View the animal from at least two positions in order to assess the body condition: standing approximately 3 metres away from the animal, facing towards its side; standing approximately 3 metres away from the animal, facing towards its tail. - The animal must meet all of the conditions to be scored as a full score. If the animal exceeds the conditions for one score, but does not meet all of the conditions for the score above, then the half score between should be used. |

**Table 1c**. Behaviour

| **Parameter** | **Assessment protocol** |
| --- | --- |
| Observer approach | - Stand 3 metres away from the animal. Before beginning the approach, allow 10-20 seconds for the animal to become comfortable with the handler. Ask the handler to hold the animal with a loose rope, and stand at arm’s length away from the animal so that it can move if it chooses. - From 3 metres away, walk at a steady, medium pace in a straight line towards the animal’s head, on the opposite side from the handler. Keep your arms still, do not carry anything in your hands, and do not make unnecessary noise whilst approaching the animal. - Approach from an angle of approximately 20 degrees. This allows the animal to see you clearly and avoids its blind spots. Do not make direct eye contact with the animal. Stop walking when you are approximately 30cm from the animal’s head. - Do not touch the animal unless it chooses to make contact with you. Observe the response to the observer, not reactions to surrounding stimuli in the environment. |
| Chin contact | - Immediately after completing the observer approach, after standing still for approximately 3 seconds, stand level with the animal’s shoulder and turn towards it until you are facing the same direction as the animal. Keeping your arm straight, move the hand closest to the animal in a smooth motion towards its chin and make gentle contact. Your hand should be lightly taking some weight, but not lifting the head. - Assess the animal’s response to the attempt to make contact. Only make one attempt to contact the chin. The animal may lift its head up or move it sideways as hand is approaching or when the chin is touched. - If the animal moves its head to avoid the approaching hand, do not pursue it. |
| Tail tuck  *(donkeys only)* | - After completing chin contact, turn towards the donkey to face the tail, and walk at a steady, medium pace around the hindquarters and along the other side, to finish beside the donkey’s head on the opposite side. - Repeat the movement in reverse to return back to where you started. Maintain a safe distance from the hind limbs whilst walking around them (at least 1 metre), and be alert to any signs that the animal may kick. - Always ensure sufficient space around the donkey to allow you to move away if necessary. Observe the donkey’s reaction as you walk towards and around the hindquarters. - If the donkey is harnessed to a cart, walk around the cart and observe the animal’s reaction as you approach the hindquarters on either side. Record not observed for all horses and mules. |
| General attitude | - Consider the way the animal has behaved throughout the entire welfare assessment; how it has reacted to its surroundings, the handler, the assessor, and being handled. - The animal may have behaved differently at different times during the assessment. - Choose the score which best describes the animal’s overall behaviour and demeanour throughout the entire time you were with it. E.g. an animal may not allow its mouth to be opened, but was calm for all other actions. Or an animal may be resistant to having the hoof picked up, but was apathetic at all other times. - A consensus should be reached between the observer and recorder on the correct score to record. |
| Spinal contact | - Stand level with the animal’s shoulder, facing the tail. Use the hand closest to the animal, and place your thumb and middle finger on either side of the animal’s spine, at the base of the mane. - Only your thumb and middle finger should make contact with the animal, at equal distance on each side of the spine, approximately 3cm on either side of the centre. Applying light downward pressure, slowly move your hand from the withers to the croup, keeping your hand in the same position throughout. - Do not pinch or squeeze the spine; the pressure applied should remain consistent from start to finish. - Repeat the same process beginning on the opposite side of the animal, using the opposite hand. - Record the strongest response which occurred on either of the two contacts. A consensus should be reached between the observer and recorder on the correct score to record. If the animal shows a Score 2 response on the first contact, it is not necessary to repeat. If the animal has wounds on the spine with fully broken skin, do not touch the wounds, and instead raise your fingers at this point whilst moving your hand along the spine. |

**Table 1d**. Lesions

| **Parameter** | **Assessment protocol** |
| --- | --- |
| Lip lesions | - Look for lesions at the corners of the lips. Look at both sides of the mouth. Record lesions of any size, using the score which describes the severity of the lesion. Do not touch the lips or open the mouth to assess. |
| Body lesions: Severity | - Body lesions are assessed by their location on the body, severity and size. Body Areas are head and ears, neck, breast and shoulders, forelimbs, ribs and flank, withers and spine, girth and belly, hindquarters, hind limbs. - Record the severity of the most severe lesion and the size of the area of skin affected by lesions of this severity. If one continuous lesion extends into more than one body area, all of the body areas affected are counted separately. - Look for lesions on the whole body of the animal, on both sides, and consider the depth of the lesions in each body area. - If the animal has more than one lesion in one body area, record the severity score of the most severe lesion. If one lesion has parts of different severity within it, record the severity score of the most severe part of that lesion. |
| Body lesions: Size | - Measure the total surface area of skin affected by lesions of the severity recorded in the same body area. If there are multiple lesions of the same severity, the areas can be combined to give an overall area range. E.g. two lesions each greater than 4 sq cm but less than 16 sq cm when combined are recorded as size Score 1 (between 4 – 16 sq cm). - If there are multiple lesions of severity Score 1, each separate lesion must be greater than the minimum qualifying size of 4 sq cm before being combined. - For Knee lesions, Tail/tail base lesions and Genital/rectal lesions size is not recorded. Record severity only. |
| Knee and Tail/tailbase lesions | - For Knee lesions and Tail/tail base lesions, size is not recorded, only severity. If the animal has more than one lesion, record the score of the most severe lesion. - If one lesion has parts of different severity, record the score of the most severe part of that lesion. |
| Genital / rectal lesions | - For Genital/rectal lesions, size is not recorded. Record severity only. |

**Table 1e**. Practice-induced conditions

| **Parameter** | **Assessment protocol** |
| --- | --- |
| Tail Mutilations | - Tail: Look at the length of the tail bone (dock). Do not be influenced by the length of the hair on the tail. If you suspect that the tail has been cut, but cannot see clearly due to the length of the hair, you can gently move the hair of the tail. - Stand in a safe position to the side of the hind limbs and be alert to signs that the animal may kick. |
| Ear Mutilation | - Look at the length and shape of the ears. Remember that donkeys and mules usually have longer ears than horses. - Record man-made cuts, slits or holes in the ears. If you do not think the damage is man-made, do not record in this category. - Do not touch the ears to assess this parameter. |
| Muzzle Mutilation | - Record man-made cuts, slits or holes in the nostrils or lips. If you do not think the damage has been caused by humans, do not record in this category. - Do not touch the muzzle area to assess this parameter. |
| Firing lesions: Severity | - Firing is the practice of burning the skin with hot irons. Evidence of firing may be seen as fresh or healed lesions or scars on any body part. Firing lesions may form linear, geometric or dot patterns. - Firing is used as a traditional method for treatment or prevention of illness or injury, and also for decoration or identification. - The location of firing on the animal’s body will vary depending upon the purpose, therefore you must check the whole body for firing lesions. - Record the severity and number of body areas affected by firing. Use the same body area divisions as for body lesions. - Look for evidence of firing on the whole body of the animal, on both sides. Remember to look at concealed areas, such as the inside of all four legs, and the underside of the belly. - Record the severity of the most severe firing lesion on the animal. If one firing lesion has parts of different severity within it, record the severity score of the most severe part of that lesion. - Do not touch the lesions or areas immediately around the lesions when assessing this parameter. |
| Firing lesions: Number of Body Areas | - Using the same body area divisions as for body lesions count the number of body areas containing firing lesions of any severity and any size. - Body areas on different sides of the animal should be counted separately. - If one continuous firing lesion extends into more than one body area, all of the body areas affected are counted separately. |
| Hobbling lesion: Severity | - Hobbling is the practice of tying ropes around the animal’s limbs as a form of restraint. - Evidence of hobbling may be seen as horizontal lesions or scars on the limbs, and may occur on the fore and/or hind limbs. Look for evidence of hobbling on all parts of all four limbs. Remember to check the back of the pasterns. - Record hobbling lesions or scars of any size, on any part of the limb. Record the severity of the most severe hobbling lesion. - If one lesion has parts of different severity within it, record the severity score of the most severe part of that lesion. If the area is not visible e.g. covered by mud or bandages, record ‘Not observed’ for this parameter. - If the area is partially covered and you can see a lesion but not the entire area, record the score of the lesion which is visible. Do not touch the lesions or areas immediately around the lesions when assessing this parameter. |

**Table 1f**. Hooves and Limbs

| **Parameter** | **Assessment protocol** |
| --- | --- |
| Gait | - Ask the handler to walk the animal in a straight line for approximately six paces, then turn round and walk back in a straight line and past you. - Ensure that the handler walks to the opposite side of the animal, and allows some loose rope so that you can see the movements of the animal’s limbs, head and neck clearly. - Observe how the animal walks. Consider whether the steps taken are even, regular and smooth. It is normal for equids to rest a hind limb, and animals doing so should still be assessed for gait. - A consensus should be reached between the observer and recorder on the correct score to record. |
| Lower limb swelling  *(Fore / hind)* | - Fore limbs: look at the flexor tendons and fetlock joints on both fore limbs from the side and from the rear. - Record any swelling which clearly distorts the straight line from the back of the knee to the fetlock joint from the side and on the inside and outside surfaces of the lower limb. - Record any other clear lumps/distortion on any part of the fetlock joint. - A consensus should be reached between observer and recorder on the correct score to record. - Hind limbs: look at the flexor tendons and fetlock joints on both hind limbs from the side and from the rear. Assess in the same way as for the fore limbs. |
| Interference lesions  *(Fore / hind)* | - This parameter is scored separately for fore and hind limbs, with the same scoring method. Interference lesions are caused by one hoof striking the adjacent limb (brushing), or by the hind hooves striking the heels of the fore limbs (over-reaching), whilst the animal is in motion. - Score lesions of any size within designated area (between the coronary band and top of fetlock joint on the inner sides of the limbs; and the back of the heels). Record the most severe lesion on either limb. - If one lesion has parts of different severity within it, record the severity score of the most severe part of that lesion. If the area is not visible e.g. covered by mud or bandages, record ‘Not observed’ for this parameter. - Do not touch the lesions or areas immediately around the lesions when assessing this parameter. |
| Hoof shape  *(Fore / hind)* | - This parameter is scored separately for fore and hind hooves, with the same scoring method. Look at the shape of both fore/hind hooves and consider the following features: toe length, heel length, straight hoof walls, hooves forming a matching pair. Record an abnormality which exists on either side of the hoof. - The hooves do not need to be perfect, but should not be so badly shaped as to impair the animal’s ability to walk, or put it at risk of pain or injury during its work. Do not be influenced by any other features of the hoof. - Do not pick up the hooves when assessing this parameter. A consensus should be reached between the observer and recorder on the correct score to record. - Only record clear abnormalities - if you are unsure, then the abnormality cannot be clear enough, and you should record Score 0. |
| Hoof quality  *(Fore / hind)* | - This parameter is scored separately for fore and hind hooves, with the same scoring method. Look at the outer surface of the hoof wall of the two fore/hind hooves and consider the quality of the hoof horn. - The wall of the hoof should be complete and intact. - Record the following features of hoof wall damage: the wall is broken or collapsed, dumping of the toe, vertical or horizontal cracks, abscess. Only record cracks in the hoof wall of approximately 2cm or more (vertically or 2cm horizontally). Only record ‘true’ cracks which penetrate through the surface of the hoof wall. Only record breakage of the hoof wall of approximately 2cm or more in length, and 1cm or more in height at any point. Ignore ragged or uneven edges in the outer 1cm rim of the hoof. Consider only the hoof horn quality and the specific abnormalities listed above when assessing this parameter. - Do not pick up the hooves when assessing this parameter. If the hooves are covered, or partially covered (e.g. with mud), and you cannot see any abnormalities, record ‘Not observed’. If the hooves are partially covered and you can see some abnormalities, record abnormalities which are visible. |
| Frog condition | - Look at the frog of both fore hooves, and consider whether there is evidence of disease. If the animal shows resistance to picking up the hoof, do not continue, and instead record ‘Not observed’ for this parameter. - If the animal has a closed shoe and you cannot see the frog, record ‘Not observed’ and make a note of this in ‘Other observations’. |
